# Supplementary figures and images for: Deletion of two-component system QseBC weakened virulence of Glaesserella parasuis in a murine acute infection model and adhesion to host cells
Source: PeerJ. 2022 Jun 24;10:e13648. doi: 10.7717/peerj.13648 (PMC9235811; doi:10.7717/peerj.13648)

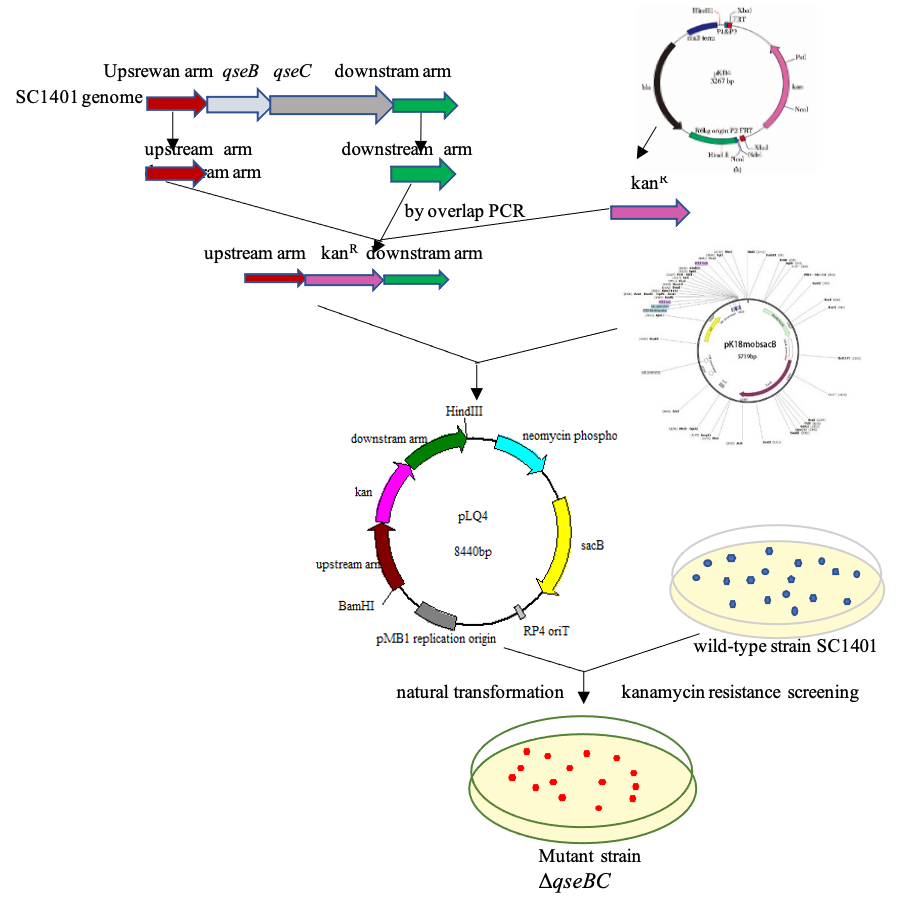

Supplement: Supplemental Information 1 [file peerj-10-13648-s001.png]
