# Supplementary material for: Deletion of two-component system QseBC weakened virulence of Glaesserella parasuis in a murine acute infection model and adhesion to host cells
Source: PeerJ. 2022 Jun 24;10:e13648. doi: 10.7717/peerj.13648 (PMC9235811; doi:10.7717/peerj.13648)
Supplement: Supplemental Information 2 [file peerj-10-13648-s002.docx]

**Adherence** **(CFU/well)**

| SC1401 | △*qseBC* | △*qseC* | C-△*qseBC* |
| --- | --- | --- | --- |
| 312×10^5^ | 165×10^5^ | 221×10^5^ | 308×10^5^ |
| 259×10^5^ | 227×10^5^ | 232×10^5^ | 312×10^5^ |
| 364×10^5^ | 144×10^5^ | 217×10^5^ | 296×10^5^ |

**Invasion (CFU/well)**

| SC1401 | △*qseBC* | △*qseC* | C-△*qseBC* |
| --- | --- | --- | --- |
| 1432 | 135 | 688 | 1087 |
| 1047 | 111 | 523 | 1173 |
| 1139 | 122 | 599 | 1003 |
