# Supplementary material for: Deletion of two-component system QseBC weakened virulence of Glaesserella parasuis in a murine acute infection model and adhesion to host cells
Source: PeerJ. 2022 Jun 24;10:e13648. doi: 10.7717/peerj.13648 (PMC9235811; doi:10.7717/peerj.13648)
Supplement: Supplemental Information 3 [file peerj-10-13648-s003.docx]

| \| Days post challenge \| \| --- \| | △*qseC* | C-△*qseBC* | mock | △*qseC* |
| --- | --- | --- | --- | --- | --- |
| 1 | 1 |  |  |  |
| 1 | 1 |  |  |  |
| 1 | 1 |  |  |  |
| 2 | 1 |  |  |  |
| 2 | 1 |  |  |  |
| 2 | 1 |  |  |  |
| 2 | 1 |  |  |  |
| 7 | 0 |  |  |  |
| 1 |  | 1 |  |  |
| 2 |  | 1 |  |  |
| 2 |  | 1 |  |  |
| 2 |  | 1 |  |  |
| 3 |  | 0 |  |  |
| 4 |  | 0 |  |  |
| 5 |  | 0 |  |  |
| 7 |  | 0 |  |  |
| 1 |  |  | 1 |  |
| 1 |  |  | 1 |  |
| 1 |  |  | 1 |  |
| 1 |  |  | 1 |  |
| 2 |  |  | 0 |  |
| 3 |  |  | 0 |  |
| 4 |  |  | 0 |  |
| 5 |  |  | 0 |  |
| 6 |  |  |  |  |
| 7 |  |  |  |  |
| 1 |  |  |  |  |
| 2 |  |  |  |  |
| 3 |  |  |  |  |
| 4 |  |  |  |  |
| 5 |  |  |  |  |
| 6 |  |  |  |  |
| 7 |  |  |  |  |
| 7 |  |  |  |  |
| 1 |  |  |  | 1 |
| 1 |  |  |  | 1 |
| 1 |  |  |  | 1 |
| 2 |  |  |  | 1 |
| 2 |  |  |  | 1 |
| 2 |  |  |  | 1 |
| 6 |  |  |  | 0 |
| 7 |  |  |  | 0 |
